# Pathways from integrated agriculture and health-based interventions to nutrition: a case from Southern Bangladesh

**DOI:** 10.1017/S1368980025000394

**Published:** 2025-08-29

**Authors:** Indu Kumari Sharma, Malay Kanti Mridha, Dirk Essink, Lalita Bhattacharjee, Victoria Fumado, Sarju Singh Rai, Mokbul Hossain, Jacqueline EW Broerse

**Affiliations:** 1 Athena Institute of the Faculty of Sciences, Vrije Universiteit Amsterdam, Amsterdam, Netherlands; 2 IS Global Institute/ Saint John of God Hospital, University of Barcelona, Barcelona, Spain; 3 International Water Management Institute (IWMI), Kathmandu, Nepal; 4 Center for Non-communicable Diseases and Nutrition, BRAC James P. Grant School of Public Health, BRAC University, Dhaka, Bangladesh; 5 Food and Agriculture Organization of the United Nations (FAO), Representation in Bangladesh, Dhaka, Bangladesh

**Keywords:** Sustainability, Nutrition outcomes, Food production, Agricultural income, Nutrition knowledge

## Abstract

**Objective:**

We aimed to analyse the effects of nutrition-sensitive agriculture (NSA) interventions on nutrition, examine the pathways within a project cycle and explore the pathways 3 years after the end of the funding period.

**Design:**

We employed a sequential mixed-methods design using (1) secondary quantitative data and (2) primary qualitative data. The quantitative data were analysed using the Mann–Whitney test, independent sample *t* test and binary logistic regression. The qualitative data were analysed thematically.

**Setting:**

This research used the case of the Integrated Agriculture and Health Based Interventions project.

**Participants:**

The quantitative data comprised 4825 households. The qualitative data included forty-six participants (twenty-eight beneficiaries and eighteen implementers) from two focus group discussions (*n* 15) and thirty-one semi-structured interviews.

**Results:**

NSA interventions reduced child underweight and improved household and women’s dietary diversity scores, breastfeeding practices, handwashing and access to Fe–folic acid during pregnancy. Pregnant and lactating women’s minimum dietary diversity increased, while children’s minimum dietary diversity reduced. Key pathways to nutrition during project implementation were food production, nutrition-related knowledge and strengthening local institutions. Sustainability of knowledge was mostly evident, followed by food production, while the strengthening of local institutions was less evident.

**Conclusions:**

Key pathways to outcomes during the project implementation were food production, nutrition-related knowledge and strengthening local institutions, as these were the main focus of the project. Income and women’s empowerment pathways could be more effective if intentionally integrated. We reiterate the need to enhance children’s dietary diversity, strengthen income–expenditure and women empowerment pathways, sustain livestock production and strengthen local institutions.

The agriculture sector has a fundamental role in addressing inadequate diets and subsequent malnutrition. The sector, through the production of diverse, nutrient-rich foods, can enhance access to nutrient-rich diets^(1–4)^. Agriculture also contributes to nutrition through the income pathway by increasing the purchasing power of farmers to make nutrition-related expenses^(1–3)^. The role is particularly vital in developing countries, where agriculture represents a significant share of gross domestic product and where high food insecurity and malnutrition persist. Therefore, it is essential to transform agriculture into nutrition-sensitive agriculture (NSA) by integrating explicit nutrition objectives and actions in agricultural interventions^(5)^. In addition, NSA interventions can improve diet and nutrition by integrating synergistic actions that translate food production or agricultural income into nutrition, such as the integration of nutrition-related education/behaviour change communication in kitchen gardens and nutrition-sensitive value chains, to name a few^(3,5)^.

Bangladesh is a lower-middle-income country that can benefit from NSA interventions in curbing undernutrition. Despite significant reductions in poverty^(6)^ and undernutrition^(7)^ over the past 25 years, Bangladesh still experiences high levels of undernutrition, particularly among children and women^(8)^. The prevalence of stunting, being underweight and wasting in children under 5 years of age is 31 %, 22 % and 8 %, respectively^(9)^. At the same time, the country has high levels of micronutrient deficiency among children and women. For instance, more than one-third (36·7 %) of women of reproductive age are anaemic^(8)^. NSA could play a crucial role in addressing these issues in Bangladesh, as 87 % of the population in the country depends on agriculture for at least part of their income^(10)^.

The government of Bangladesh recognised the role of agriculture in nutrition following the 1996 World Food Summit^(11)^. The formulation of the National Food Policy (2006), the National Food Policy Plan of Action (2008–2015) and the Bangladesh Country Investment Plan on Agriculture, Food Security and Nutrition are important milestones that focus on the role of agriculture in nutrition^(11)^. Likewise, nutrition sensitivity in agriculture is explicitly reflected in the National Nutrition Policy 2015, Second National Plan of Action for Nutrition (NPAN 2) (2016–2025), National Food and Nutrition Security Policy 2020, National Food and Nutrition Security Plan of Action 2020–2030 and Agriculture Policy 2019. Other agricultural sectors, such as food, fisheries and livestock, also have integrated nutrition objectives and actions in their road maps and plans of action. Although Bangladesh has seen progress in producing diverse nutrient-rich foods^(12)^, food production is still heavily dominated by rice, with inadequate production of non-staple foods that promote dietary diversity^(12)^. Even in situations with enough production of diverse foods, it may not necessarily lead to dietary diversity due to a lack of knowledge or selling of the product due to the prioritisation of non-nutrition-related expenditure. Building on the remarkable declines in undernutrition over the last decades in Bangladesh, accelerated efforts to implement comprehensive nutrition-sensitive policies can help to meet the national nutrition targets and pave the way for sustainable food systems. There is, thus, an increasing need to strengthen and accelerate the implementation of NSA to improve diets and nutrition.

Research on agriculture-to-nutrition linkages in Bangladesh has been apparent since 2000^(13)^, ranging from literature reviews^(6,14)^ to empirical studies. Empirical studies center on agricultural factors affecting nutritional status^(7,15)^, agriculture to nutrition pathways and the effects of specific NSA interventions such as home garden and aquaculture on nutrition^(10–12,16,17)^. Likewise, a review identified four pathways from agriculture to nutrition—food production, agricultural income, policy and food price and women’s empowerment^(6)^, highlighting gaps in intervention impacts on diets and pathways, particularly income and women’s empowerment. Also, existing studies heavily focus on agriculture interventions over NSA and lack studies on sustainability. Most focus on project implementation, with limited research on sustainability. For instance, one study reported sustained effects of a nutrition-sensitive home garden intervention on vegetable consumption^(16)^. Studies assessing the impact of NSA interventions on nutrition, their pathways during the project period and the sustainability of these pathways beyond project funding are insufficient.

This study aims to analyse the effects of NSA interventions on nutrition outcomes and the pathways to nutrition during the project cycle and examine the pathways sustained 3 years after the end of the funding cycle. The study used the Integrated Agriculture and Health Based Interventions (IAHBI) project as a case. The project employed evidence-based nutrition interventions in the agriculture and health delivery services in Southern Bangladesh (2012–2015) to improve household food security and nutritional status, with a focus on mothers and young children^(18,19)^. The evidence generated from this case study will assist agricultural interventions in enhancing their contribution to improving nutrition in the context of Bangladesh and similar low and middle income countries, as the evidence of the sustainability of the interventions beyond their funding cycle is limited at the global level^(2,20)^.

## Study methodology

### Study design

We employed a case study approach with a mixed-methods design^(21)^, conducted sequentially with a quantitative phase, followed by a qualitative phase. The case used in this research was the IAHBI Project. The case study approach was chosen because it allows for the exploration of complex interventions and programs^(22)^, making it well-suited to study the IAHBI Project.

Quantitative methods allowed us to assess the effects of interventions on nutrition outcomes as reported during the end of the project cycle through the analysis of baseline and endline survey data. The qualitative approach was used to examine two aspects: (1) the pathways during the project cycle and 3 years after the project funding period and (2) food consumption practices 3 years after the end of the project cycle. Three years were chosen to allow a sufficient period to capture the sustained pathways. The quantitative and qualitative methods helped construct impact pathways from IAHBI Project interventions to nutrition outcomes.

The quantitative component of the study comprised secondary data from (1) the baseline survey conducted from late 2013 to early 2014 by BRAC James P. Grant School of Public Health, BRAC University and the Institute of Nutrition and Food Science^(23)^ and (2) the endline survey carried out from January to February 2016 by the Institute of Nutrition and Food Science, University of Dhaka, Bangladesh^(24)^. The qualitative data were collected from October 2018 to January 2019 through focus group discussions (FGD) and semi-structured interviews (SSI) with project implementers and beneficiaries.

### Setting

The study was conducted in Southern Bangladesh. The quantitative data covered five sub-districts of the project, namely Dacope and Koyra of Khulna district, Muladi of Barishal district and Assasuni and Shyamnagar of Satkhira district. From these sub-districts, we selected the Dacope sub-district to collect qualitative data at the field level and included data from national and district levels. Southern Bangladesh has been affected by repeated devastating cyclones, for instance, cyclone *Sidr* in 2007^(25)^ and *Aila* in 2009^(26)^. Such disasters have adversely affected the livelihoods of thousands of populations by further pushing them into poverty and reducing access to resources such as food. Similarly, children under 5 years and lactating mothers in Southern Bangladesh also experience limited quantity and variety of food consumption^(25)^.

### Project interventions

The IAHBI Project is a multisector project funded by the United States Agency for International Development, implemented from September 2012 to September 2015^(18,19)^. The project was implemented by the FAO of the United Nations and the United Nations Children’s Fund in collaboration with the Government of Bangladesh partners and an NGO partner named Sheba Manab Kallyan Kendra^(18,19)^. While the Ministry of Fisheries and Livestock was the leading government organisation, other government partners were the Ministry of Health and Family Welfare, the Ministry of Agriculture and the Ministry of Food and the Ministry of Public Administration^(27)^.

The project comprised agriculture interventions and nutrition activities. The agricultural interventions included technologies and inputs for three sub-sectors: horticulture, livestock and aquaculture. The horticulture component focused on vegetable production training and supplies, such as saplings of fruits, vegetable seeds, fertilisers and farming tools. The livestock component involved training on improved production technologies and inputs for small livestock. The inputs mainly included goats/sheep, chickens and ducks and feed and shelter. Within the aquaculture component, the project trained the beneficiaries on fish cultivation and distributed supplies, such as fingerlings, lime, feeds and fertilisers. Nutrition and Water, Sanitation and Hygiene (WASH) were central components integrated into the agricultural interventions to enhance the nutrition sensitivity of the interventions, focused on behaviour change activities through awareness sessions, healthy cooking demonstrations and training on nutrients and food preservation^(18)^. The project formed farmer field schools (FFS) to deliver interventions where the women farmers with similar interests engaged in meetings, learning and demonstrations on food production and nutrition^(18)^. The FFS members later provided cascading training to members of women farmer groups^(18)^. The project also incorporated nutrition-specific activities on enhancing the uptake of Fe–folic acid (IFA) supplementation for pregnant women and deworming for children 6–23 months old^(18,28)^.

### Participants and recruitment

The quantitative research included four categories of survey populations: women of childbearing age, mothers or caregivers of children under 5 years of age, pregnant women and lactating women^(29,30)^. Both surveys used a two-stage cluster sampling to provide a representative sample per union council according to their population size. For the baseline survey, 4–8 clusters with homogenous characteristics of households were randomly selected per union council^(30)^. In the second stage, twenty-four households per cluster were selected, totalling 1536 households^(30)^. For the endline survey, the first stage involved selecting seventy-five villages through probability proportional to size^(29)^. In the second stage, forty-three households were systematically sampled in each village, covering a total of 3289 households^(29)^.

The baseline and endline surveys comprised 1536 households and 3289 households, respectively^(29,30)^. The sample sizes included for the secondary data analysis in this study were calculated after deleting missing and non-applicable cases. We analysed the secondary data on undernutrition for three population groups: children (0–23 months and 0–59 months), pregnant women and lactating women.

The participants of the qualitative research were either project implementers or project beneficiaries. The project implementers further represented two groups, direct implementers and community members involved in the implementation. We obtained the initial list of the participants from the project implementer (s) followed by the snowball method to recruit the participants. The implementers had to represent the national, district or sub-district and Union Porishad levels. The details are explained in another paper that used the same qualitative methodology^(19)^. The participants are identified in the quotes as the following labels^(19)^:

*IN-Implementers at the national level; ID-Implementers at district and sub-district level; IUP-Implementers or members involved in implementation at Union Porishad; BL-Beneficiary- Farmer Field School (FFS) leaders; BM- Beneficiaries-FFS and women farmer groups members; BF- Beneficiaries- Focus Group Discussion (FGD)*

### Data collection

The quantitative component of the research used secondary data from the project baseline and endline surveys. Key variables were selected from these surveys (see variables section), through some topics had differing options/fields between the two. Online supplementary material, Supplemental 1 illustrates the broad topics covered in the surveys^(29,30)^. Data were collected through interviews and anthropometric measurements, with methods varying by nutrition outcomes. Undernutrition in children was assessed using anthropometric data (height and weight) and micronutrient status (Hb levels for anaemia). For pregnant women, undernutrition was measured using mid-upper arm circumference (MUAC) and Hb levels. Dietary diversity was assessed via a 24-hour recall, while household surveys collected data on IFA intake during pregnancy, breastfeeding and WASH practice levels to examine anaemia.

Qualitative data from beneficiaries were collected using FGD and SSI, while the data from the implementers were collected using only SSI. The FGD and SSI guidelines explored project participation, perceived effects on nutrition and the barriers and facilitators in regard to implementation and sustainability. The tools can be accessed from a past study on the factors affecting the implementation and sustainability of NSA interventions^(19)^. Two trained research assistants collected qualitative data under supervision from a senior researcher. While two research assistants, supervised by a senior researcher, collected the qualitative data. FGD were conducted by both assistants, while at least one assistant guided the SSI. The interviews and FGD lasted 20–70 min and 51–62 min, respectively. The data were tape-recorded with participants’ consent and transcribed by the research assistants. As suggested by a past study^(23)^, we determined the number of participants after data saturation, defined as when no new codes on the effects and pathways emerged.

We regularly reviewed the quality of data during the research process. The quantitative secondary data utilised in our analysis had undergone prior cleaning and quality checks conducted by the evaluation team. For qualitative data, we adopted a comprehensive approach by gathering insights from multiple stakeholders, including community members and program implementers. The quality assurance further involved regular debriefing sessions during data collection, rigorous analysis techniques and subsequent dissemination and review of findings by senior researchers.

### Variables included in the quantitative data

The dependent variables were outcomes on nutrition, WASH and antenatal care visits. Change in these outcomes was assessed by looking at the difference in these effects across baseline and endline. The independent variable was the group as baseline and endline (see Box 1 for details).

**Box 1.** 

Nutrition included four categories of variables as follows: The *undernutrition* was analysed using anthropometric measurements and micronutrient deficiency. Anthropometric measurements in children under 59 months of age included stunting, being underweight and wasting. These were measured using the WHO classification by applying a z-score less than –2 for height-for-age (stunting), weight-for-height (wasting) and weight-for-age (underweight)^(31)^. The variable on micronutrient deficiency in children was anaemia, defined as having Hb levels less than 11 g/dl. Undernutrition in pregnant and lactating women was assessed using MUAC and Hb. While having a MUAC of less than 23 cm in pregnant and lactating women,^(32)^ the cut-off values of Hb for calculating anaemia were 11 g/dl and 12 g/dl for pregnant women and lactating women, respectively^(33)^. The assessment of *dietary diversity* included continuous and binary variables. The continuous variable was the dietary diversity score (DDS), measured at the household and individual levels for children, pregnant women and lactating women. The household DDS, children’s DDS and women’s DDS were computed as a total score out of 12^(34)^, seven^(35)^ and nine^(34)^ food groups, respectively. As the questions applied at the endline for household and pregnant and lactating women included more options for food items, we matched the number with the baseline using a guideline by FAO^(34)^. The binary variable was minimum dietary diversity (MDD), defined as meeting at least four out of seven food groups for children 6–23 months^(36)^ and at least five out of the nine food groups for pregnant^(34)^ and lactating women. *Breastfeeding* practice was measured using two variables. (1) The early initiation of breastfeeding is defined as the initiation of breastfeeding within 1 hour of birth. (2) Exclusive breastfeeding is described as feeding breastmilk exclusively until the child is six months old^(36)^. *Intake of IFA included* a yes–no questions about whether lactating women, during their last pregnancy, received IFA. The WASH included four variables: access to an improved sanitation facility, access to an improved drinking water source, drinking water purification and handwashing with soap during critical times. The baseline survey included the following improved sources of sanitation facilities: piped sewer system, septic tank, ring slab with water seal and pit latrine with slab^(30)^. The endline comprised the following facilities as improved: flush through the pipe for discharge, flush to a septic tank, flush to pit latrine, ventilated improved pit latrine and pit latrine with slab filtering^(29)^. The unimproved sanitation facilities included in the baseline were ring slab without water seal, pit latrines without a slab, hanging latrines, no facility or others^(30)^. Likewise, the endline survey comprised the following unimproved sources of sanitation facility: flush to non-septic tank, flush to discharge elsewhere, flush but don’t know where filth is, pit latrine without slab/open pit latrine, composite latrine, hanging latrine, no facility or others filtering^(29)^. The baseline survey included the following improved sources of drinking water: piped water to the dwelling yard or public tap; tube well or borehole that is shared or at the household and protected dug well^(30)^. The improved drinking water source included in the endline survey was piped water to the house, courtyard or neighbours’ house, public tap, deep tube well or own or joint or well with others’ ownership; protected dug well; rainwater collection and pond sand filtering^(29)^. Unprotected wells, surface water and tanker water were considered unimproved sources. *The handwashing practices* used a yes–no question that covered handwashing practices with soap seven times—before preparing food, before eating, after eating, before feeding a child, after disposal of the child’s faeces, after cleaning the child’s anus and after defecation^(29,30)^.

### Data analysis

The analysis of this mixed-methods design involved three steps. During the first step, we analysed the quantitative data to calculate the effects of the interventions on the outcomes described in the previous section, using IBM SPSS Statistics 27. We applied three statistical tests to report the differences in the indices across baseline and endline surveys: the Mann–Whitney test for non-normally distributed continuous dependent variables, the independent sample *t* test for normally distributed continuous dependent variables and binary logistic regression for categorical dependent variables. We interpreted the results of binary regression using OR. A *P*-value of less than 0·05 was considered statistically significant. We calculated the frequencies, percentage, sd and mean values using descriptive analysis.

The second step involved the analysis of qualitative data, using ATLAS Ti version 8.5. The focus of the qualitative data was on the pathways. The data were analysed using a thematic analysis employing a recently published framework^(3)^ against the following five pathways from NSA interventions to nutrition outcomes:

- Food production: production of nutrient-rich foods to increase household access and consumption.
- Agricultural income: increase in agricultural income that can enhance the affordability of food and nutrition-related expenses such as purchasing nutrient-rich foods
- Nutrition-related knowledge: increase in knowledge of nutrition and WASH practices and behaviour change
- Women empowerment: empowerment of women and acknowledgement of their role in nutrition
- Strengthening of local institutions: strengthening of institutions to enhance agriculture and health service delivery.

The strengthening of local institutions in this context refers to initiatives aimed at improving service delivery within subnational institutions, particularly in the health and agriculture sectors. The objective is to build the capacity of these institutions to effectively contribute to improved nutrition outcomes within their operational areas.

In the context of the IAHBI project, three key strategies were employed to strengthen institutions:

1. Integration of antenatal care services, Fe supplementation and counselling for antenatal check-ups within health extension services
2. Enhancement of access to agriculture (crop, livestock and fishery) by providing vaccinations, training on food production techniques and supplying inputs such as seeds to communities
3. Establishment of FFS as a vital platform for enhancing knowledge sharing, technology uptake and capacity building among communities

The project interventions were implemented jointly to create synergies between agriculture and health, fostering multisectoral collaboration. This approach focused on integrated nutrition training and devolving responsibilities to different levels to empower communities, the government and the private sector^(18)^. These activities aimed to improve the overall capacity and effectiveness of local institutions in promoting nutrition and agriculture-related services and outcomes (see online supplementary material, Supplemental file 2).

Of these pathways, the IAHBI Project mainly focused on food production, nutrition-related knowledge and behaviour change and strengthening of local institutions. An increase in income was also partly covered by its interventions, whereas women’s empowerment covered their empowerment in terms of access to the selling of agricultural products. We analysed the pathways across two periods of time: pathways experienced during the project funding cycle and the pathways sustained 3 years after the end of the project.

During the third step, the results of the quantitative and qualitative components were integrated^(21)^. A framework published in a past study was applied to first describe the effects of interventions followed by the pathways, resulting in overall impact pathways.

## Results

This section comprises three sub-sections—participants’ characteristics and interventions received by the beneficiaries, effects of NSA interventions on nutrition and the pathways to the outcomes.

### Participants characteristics

The mean age of children in baseline and endline surveys was, respectively, 28·26 (17·03) and 29·65 (16·46) months, and the male–female distribution among the children was almost equal. Most of the participants identified as Muslim by religion and lived in Muladi sub-division (see Table 1 for details).

**Table 1.** Socio-demographic and economic characteristics of the participants on baseline and endline survey

Socio-demographic and economic characteristics	Baseline		Endline	
	Mean	sd	Mean	sd
Age of < 5 years child in months	*n* 633		*n* 1220	
	28·26	17·03	29·65	16·46
	*n*	%	*n*	%
Sex of child	*n* 633		*n* 1220	
Male	324	51·2 %	601	49·3 %
Female	309	48·8 %	619	50·7 %
	Mean	sd	Mean	sd
Religion	*n* 1536		*n* 3289	
Muslim	1148	74·7	2671	81·2
Hindu	388	25·3	618	18·8
Geographical location				
Total households	*n* 1536		*n* 3289	
Assasuni	300	19·5	575	17·5
Shyamnagar	312	20·3	572	17·4
Dacope	300	19·5	699	21·3
Koyra	312	20·3	658	20·0
Muladi	312	20·3	785	23·9

Abbreviations: *n*, total number.

The participants of the qualitative data included forty-six people, of whom twenty-eight were beneficiaries and eighteen were implementers. The beneficiaries were women and represented either FFS or women farmer groups. The implementers represented national (2), district or sub-district (12) and Union Parishad (4).

#### Interventions received by the beneficiaries

The beneficiaries who participated in qualitative data collection stated that they were part of at least one agricultural production and nutrition education/behaviour change communication intervention (see online supplementary material, Supplemental file 2). A few beneficiaries also received extension services on nutrition-specific interventions and/or vaccination support for livestock.

### Effects

This section describes the effect on nutrition (and WASH) at endline as well as those reported 3 years after the end of the project cycle by the qualitative data. Table 2 and Table 3 illustrate the effects based on quantitative data, categorised across three outcomes, nutrition, WASH and antenatal care.

**Table 2.** Change in nutrition outcomes for categorical variable

Outcomes	Baseline (%)	Endline (%)	OR/ Exp. (B^*^)	95 % CI	*P*
Nutrition					
Children 0–59 months; N: B = 633; E = 1220					
Stunting	34·3	31·9	0·897	0·73, 1·10	0·297
Underweight	30·3	22·8	0·678	0·546, 0·841	**< 0·001**
Wasting	11·1	9·8	0·869	0·636, 1·188	0·379
Children 6–23 months					
Anaemia (Hb < 11 g/dl); N: B = 185; E = 338)	67·6	63·9	0·850	0·581, 1·242	0·401
Children 0–6 months N: B = 70; E = 117					
Exclusive breastfeeding	40	69·2	3·375	1·818, 6·266	**< 0·001**
Children 0–23 months					
Early initiation of breastfeeding N: B = 275; E = 527	72·7	81·0	1·601	1·136, 2·257	**0·007**
Children 6–23 months					
MDD (≥ 4 food groups out of 7), N: B: 205; E = 410	31·9	21·7	0·589	0·405, 0·858	**0·005**
Pregnant women					
Underweight (MUAC < 23 cm); N: B = 99; E = 248	21·2	19·8	0·915	0·512, 0·515	0·761
Anaemia (Hb < 11 g/dl); N: B = 91; E = 226	61·5	57·1	0·831	0·505, 1·367	0·467
MDD (≥ 5 out of 9); N: B = 99; E = 255	27·3	51·4	2·817	1·699, 4·672	**< 0·001**
Lactating women					
Underweight (MUAC < 23 cm); N: B = 272; E = 511	26·8	22·9	0·810	0·577, 1·135	0·221
Anaemia (Hb < 12 g/dl); N: B = 159; E = 384	66·0	53·9	0·601	0·409, 0·884	**0·010**
MDD (≥ 5 out of 9); N: B = 272; E = 518)	27·2	59·7	3·937	2·861, 5·419	**< 0·001**
ANC check-ups during pregnancy; N: B = 272; E = 523	68·8	73·6	1·268	0·919, 1·749	0·148
IFA supplementation during last pregnancy; N: B = 272; E = 523	57·70	73·0	1·984	1·458, 2·702	**< 0·001**
WASH					
Improved drinking water source; N: B = 1536; E = 3286	57·2	81·8	3·403	2·975, 3·892	**< 0·001**
Improved sanitation facility(latrine) N: B = 1536; E = 3262	57·2	85·1	4·291	3·732, 4·934	**< 0·001**
Purification of drinking water; N: B = 1536; E = 3289	21·5	22·8	1·078	0·931, 1·248	0·317
Handwashing with soap: B = 1536; E = 3286					
Never	3·6	1·7	0·475	0·327, 0·692	**< 0·001**
Before preparing food	30·6	51·0	2·364	2·079, 2·687	**< 0·001**
Before eating	45·2	63·5	2·109	1·865, 2·385	**< 0·001**
After eating	17·8	17·8	0·995	0·850, 1·166	0·955
Before feeding a child	3·8	8·0	2·169	1·624, 2·896	**< 0·001**
After disposal of child faeces	6·0	9·7	1·688	1·327, 2·146	**< 0·001**
After cleaning the child’s anus	7·1	7·1	0·995	0·785, 1·259	0·964
After defecation	84·7	90·4	1·692	1·412, 2·027	**< 0·001**

* N represents the total sample included ANC, antenatal care, B, baseline, E, endline; Exp (B) refers to OR; IFA, Fe–folic acid; MDD, minimum dietary diversity; MUAC, mid-upper arm circumference; N, total number.

**Table 3.** Change in nutrition outcomes for continuous variable

Outcomes	Total sample							
	Mean, baseline	sd	Mean, end line	sd	*P* ^†^	Z	*P* ^‡^, mean difference	t
Nutrition								
HDDS; N: B = 1536; E = 3289	6·80	1·542	7·367	1·45	**< 0·000**	–11·96		
Children’s DDS; N: B = 205; E = 410	2·79	1·40	2·548	1·32	0·059	–1·890		
Children’s Hb (6–23 months) N: B = 185; E = 338)	10·28	1·299	10·353	1·354	0·308	–1·019		
Pregnant women’s DDS; N: B = 99; E = 255	3·707	1·44	4·643	1·243	**< 0·001**	–5·619		
Pregnant women’s MUAC; N: B = 99; E = 248	24·692	2·2829	25·431	2·7377	**0·027**	–2·210		
Pregnant women’s Hb; N: B = 91; E = 226	10·457	1·333	10·649	1·3016			0·239, −0·1920	1·180
Lactating women’s DDS; N: B = 272; E = 519	3·66	1·359	4·836	1·192	**< 0·001**	–11·322		
Lactating women’s MUAC; N: B = 272; E = 511	24·29	2·288	25·111	2·8048	**< 0·001**	–3·883		
Lactating women’s Hb; N: B = 159; E = 384	11·42	1·35	11·767	1·387	**0·008**	–2·657		

ANC, antenatal care; B, baseline; DDS, dietary diversity score; E, endline; HDDS, household dietary diversity; MUAC, mid-upper arm circumference; the values in bold represent statistically significant p-values.

† *P*-value and Z score derived from Mann–Whitney test result; N, total number.

‡ *P*-value and Z score derived from independent sample *t* test results.

#### Effects on nutrition

This section presents the effects of the intervention during the project implementation analysed using the quantitative data, integrated with qualitative findings.

#### Undernutrition

The prevalence of underweight among children under five years of age decreased significantly at the endline. The children at the endline were 32 % less likely to be underweight than those at the baseline (OR: 0·678; 95 % CI: 0·546, 0·841). However, the stunting and wasting rates did not differ significantly.

Among pregnant and lactating women, the mean score of MUAC increased significantly from baseline to endline. The increase in the mean MUAC for pregnant and lactating women from baseline to endline was from 24·69 cm (2·28) to 25·43 cm (2·73) and 24·29 cm (2·28) to 25·11 cm (2·80), respectively. However, the decrease in the proportion of underweight women was not statistically significant. Anaemia decreased significantly from baseline to endline in lactating women (OR: 0·601; 95 % CI: 0·409, 0·884) but not in pregnant women.

No qualitative data were collected to study the effects of undernutrition.

#### Dietary diversity

Compared with the baseline, mean household DDS was higher at the end line with a statistically significant difference (P < 0·001). The DDS at baseline and endline were 6·80 (1·54) and 7·37 (1·40), respectively.

Qualitative data collected 3 years after the end of the project funding cycle reveal that household members, in general, included more diverse foods in their meals, particularly vegetables, as a result of the project intervention. Meat was consumed occasionally, often during *the visit of guests*. Consumption of milk was limited due to the lack of cattle or money, or due to cultural preferences of avoiding sheep’s milk.

In the case of children, the proportion of MDD decreased significantly from baseline to end line (OR = 0·589; 95 % CI: 0·405, 0·858). There was no statistically significant difference in children’s mean DDS.

Qualitative data collected 3 years after the end of the project cycle indicate several improvements. Beneficiaries provided extra food to children by adding diverse foods such as vegetables, fish and eggs in *jaw/khichuri,* mixed porridge with rice and lentils. Milk consumption by children was better compared with other household members, as a few participants stated having bought milk for their children. The following citation underscores the improvement:

*‘To be very honest, their food habit has improved a lot […] Previously, they did not give any extra food to their infants who were older than six months. But now they are preparing Khichuri with extra vegetables for their babies. Khichuri [a sub continental dish prepared by mixing vegetables, pulse, rice] is nowadays being made with eggs as an additional thing.’* [IUP2]

The consumption of nutrient-rich foods among children was affected by a lack of money and the consumption of packaged processed food with high sugar content [*tea biscuit, tiger drink*] accessed from the local market. The following quote of a beneficiary highlights the role of money/income in access to a diverse diet.

*‘[They taught us to cook food in a nutritious way, for example], boiling the fish, and expander the bones, to make a round food, adding spice to make fish balls then they gave us to eat, we ate it, and it was tasty [She was happy to share this]. We need those things to make it at home, but we are poor […]. Whoever can earn they eat that way.’* [BM6]

Among women, mean DDS increased significantly from baseline to end line for pregnant women and lactating women from 3·78 (1·44) to 4·64 (1·243) and 3·66(1·359) to 4·836(1·198), respectively. The proportion of women reaching the MDD also was more than doubl for pregnant women (OR: 2·817; 95 % CI: 1·699, 4·672) and was more than triple for lactating women (OR: 3·937; 95 % CI: 2·861, 5·419).

The qualitative data are in alignment with improved dietary practices among pregnant women. During pregnancy, beneficiaries reported having consumed diverse foods such as meat, fish, egg, milk and vegetables. However, an FFS leader indicated that the change in food intake during pregnancy was less than needed.

#### Intake of iron–folic acid

The odds of taking IFA during pregnancy were 1·98 times more at the end line than at the baseline (OR: 1·984; 95 % CI: 1·458, 2·702). According to the qualitative data, pregnant women during the project funding cycle received Fe supplementation and antenatal check-ups.

*‘Every month, they used to give this [iron folic acid] and yes [we ate]. Now also they give, but we don’t go to bring. If we go, they give, those who work for government works, like those who give vaccines.’* [BF2]

After the project cycle, they had to utilise the supplements from health centres, which were convenient for some and too far for a few other participants.

#### Breastfeeding practices

Children at the end line were 1·6 times more likely to receive early breastfeeding and 3·3 times more likely to be exclusively breastfed than the children at the baseline (95 % CI: 1·136, 2·257 and 1·818, 6·266, respectively). Qualitative data indicate that the beneficiaries started breastfeeding their children within 1 hour of birth. Exclusive breastfeeding practice improved, but the results were mixed as two out of three beneficiaries had either stopped breastfeeding or provided water before the child completed 6 months.

#### Effects on WASH

Several WASH practices saw improvements (see Table 2). Compared with the baseline, the households at the endline were more likely to access improved sanitation by 4·29 times (OR: 4·291; 95 % CI: 3·732, 4·934) and access to improved drinking water sources by 3·403 times (OR: 3·403; 95 % CI: 2·975, 3·892). Likewise, washing hands with soap increased significantly from baseline to endline: before preparing food, before eating, before feeding a child, after the disposal of child faeces and after defecation.

Qualitative data also showed sustained improved sanitation and hygiene practices and fewer cases of diarrheal diseases. The IAHBI project imparted knowledge of sanitation and hygiene, and the demonstration of *tipi-tap* ^*1*^, along with the sanitation facilities built by other collocated projects, has helped improve access. The project also improved practices of washing hands with soap before eating and after going to the toilet and the use of sanitation facilities. However, the lack of access to an improved source of drinking water remained a problem for a few beneficiaries as they relied on pond water, as indicated in the following quotation.

*‘Not everyone has a water tank facility. They collect the rainwater and make it pure to drink […]. We do not lead such a privileged life. What we old people drink is the same water as the children. [We do], just literally everything with pond water.’* [BM2]

### Pathways to nutrition outcomes

We analysed data across five pathways contributing to outcomes: food production, agricultural income, knowledge of nutrition and WASH, strengthening of local institutions and women empowerment. First, we present the effects on the five entry points of the pathways, followed up by a section on how these contributed to nutrition outcomes.

#### Food production

During the project implementation, inputs, training on production practices and knowledge of food safety were provided, leading to improved production of diversified foods and increased yields. This increase in diversity and amount of food produced enhanced household access to food, a crucial pathway toward improving food consumption.

The beneficiaries mainly applied seeds and techniques to produce food, for example, preparing beds, fertilisers and pesticides, thereby increasing the quantity and quality of vegetables. The project also increased livestock ownership through inputs, training and vaccination. The beneficiaries possessed chickens, ducks and eggs, while a few still had those due from the project. For aquaculture, some beneficiaries reported having applied the fish fingerlings and the techniques provided by the project, such as grounding fish, maintaining the pond and preparing food. There was an increase in varieties of fish, for instance, by the introduction of *tilapia.* The lack of land and the death of livestock were two main impediments to production. The following quotes exemplify the increase in production and subsequent increase in food access and consumption, respectively:

*‘Cultivation increased because we gave the fertilizer, we cultivated by making a bed, and we cultivated more than one seedling.’* [BM6]

*‘I received vegetables support and hens from the project…. we raised ducks and hens at home because buying eggs at 10 taka each for daily consumption by the children was [costly], and eggs provide extra calorie. We needed to ensure they had enough food, which is why we raised ducks and hens at home.’* [BM8]

A few beneficiaries also reported continuation of production practices such as bed-making and applying fertilisers for vegetable production and fish-rearing techniques 3 years after the funding cycle. A few also indicated the application of poultry production techniques. Participants stated that the fruits from its saplings were not produced during the project but started only after a couple of years. Livestock sustainability was affected by the selling or death of livestock, especially poultry. They cited the following reasons for death: lack of food or shelter, diseases, or salty water in the case of poultries. A couple of beneficiaries also used coping mechanisms against livestock death, for instance, selling their poultry before the water turned salty. According to a beneficiary,

*‘They gave eight hens. Hens were laying 5–6 eggs every day. In the flooding, saltwater came, they drank that salty water, and they died […] Out of 8 ducks, two died. We ate some, some were lost. Most of the time in our pond, when the salty water comes, ducks don’t stay alive. […] Now, in the sweet [fresh] water, the ducks gave 11 babies. So, when the salt water comes, before that I sell them. […] Yes. I sold all.’* [BM8]

Nevertheless, a small number of beneficiaries indicated the sustainability of livestock interventions:

*‘It is not correct to say all died because, in some households, the ducks are giving birth and continuing production.’* [BF1]

Dissemination of knowledge and techniques is also noteworthy. A couple of beneficiaries reported spillover effects as they applied the production techniques to produce rice, fish in existing ponds or crabs. Other projects and initiatives also facilitated the enhancement or continuation of the production techniques, as indicated in the following quote.

*Here, almost all families cultivate vegetables and spinach nicely. After going to each training, people had an experience. I made this [vegetable] bed, I knew as they were telling us in the training from the [IAHBI] Project, after that, they also informed from World Vision, Care Bangla.”* [BM8]

### Income/selling

The project contributed to increased income through enhanced food production, alongside increased awareness of selling surplus commodities. Consequently, there was a decreased reliance on purchasing food and fertilisers due to increased self-production. While this led to a moderate rise in food expenditure, the outcomes varied because some beneficiaries chose to allocate resources towards other household necessities, like spending money on children’s education, rather than focusing solely on nutrition.

Increased production has reduced cost by decreasing the need to purchase vegetables, eggs, fertilisers and pesticides. Some beneficiaries also earned income by selling vegetables, poultry/eggs, livestock, fish and crops. The following quotes illustrate an increase in sales or income:

*‘Earning up to 500 takas/time for spinach after using a raised bed system’* [BL2].

*‘They [project] were concerned about both nutrition and income. Their priority was to fulfil their [people’s] demand for nutrition, and the excess products were sold in the market. They consumed about 30 % of the eggs […] the beneficiaries could have sold around 70 % of eggs produced.’* [ID1]

Beneficiaries typically sold vegetables on a small scale to households that had finished their harvest, didn’t grow vegetables or during lean or the rainy seasons. A few sold large quantities to merchants. Income from livestock mainly came from selling eggs, poultry (ducks and hens) and, to a lesser extent, goats and sheep. While some beneficiaries were already selling aqua products in bulk before the project, a few others began doing so due to the project’s influence.

Production income was partly sustained as several beneficiaries continued selling vegetables, poultry or fish 3 years after the funding cycle. Many also had other livelihoods besides project income, including selling homegrown products (hen/ducks, eggs, rice and dried beans and aqua products), natural resources (crabs and fish from the forest or canals) or earning wages from farm or fish hatchery. Additionally, many households relied heavily on non-agricultural income sources like metalwork, daily labour, furniture shops and remittance from labour in India.

### Knowledge of nutrition and water, sanitation and hygiene

Throughout the project, increasing knowledge of nutrient-rich food and benefits (food safety) of chemical-free crops and vegetables enhanced the consumption of diverse foods. Household production of nutrient-rich foods also increased due to education on nutrient sources and food safety practices.

The project improved knowledge on at least one of these topics: diverse food consumption, food consumption and care practices for special groups and WASH. Beneficiaries recognised the importance of nutrient-rich foods such as meat, fish, eggs, legumes, vegetables, spinach, fruit and milk. Some understood the importance of micronutrient-rich foods (Fe, Ca, vitamins), micronutrient deficiencies (e.g. Vitamin A) and the value of small fish consumption. They also learned nutrition-sensitive cooking techniques, such as washing vegetables before cutting, covering pots while cooking and avoiding high heat. A few beneficiaries demonstrated awareness of food consumption and care practices for children, pregnant and lactating women and adolescent girls. They knew at least one infant and young child feeding (IYCF) practice, such as initiating breastfeeding within one hour of birth, exclusive breastfeeding for 6 months and appropriate complementary feeding. Beneficiaries were also aware of the additional food and care required during pregnancy. They were aware of the consumption of nutrient-rich foods such as Fe (spinach and liver) and Ca (dairy products), Fe supplements, the importance of proper rest and care and regular health check-ups during pregnancy. The WASH-related topics included handwashing with soap, sanitation (construction and use of toilets) and the use of safe drinking water. The following quote highlights the knowledge gained and the translation of knowledge into food consumption, respectively.

*I learned that you should provide adequate food for pregnant mothers and children. Pregnant women should eat foods like spinach, vegetables, small fish, egg, pulses, and fruits. […]. We learned from the project that we should wash our hands and only then eat.”* [BM6]

*I have a 2-year-old child, during pregnancy, I was eating mostly vegetables as they have many vitamins, and also fish, meats, eggs, milk.* [BF2]

The knowledge was reported three years after the funding cycle and thus sustained. It is noteworthy that some beneficiaries also acquired knowledge from other sources such as schools, family members and other similar projects implemented after the project, thus facilitating knowledge retention.

### Strengthening of local institutions

The project strengthened existing institutions of health and agriculture during implementation. Beneficiaries reported that the project increased access to antenatal care services, Fe supplementation and counselling for antenatal check-ups, all of which are vital mechanisms linked to improving nutrition. These services encompassed nutrition-specific interventions on micronutrient supplementation and counselling to pregnant women on proper nutrition practices. Concerning agriculture, the project enhanced access to livestock vaccination services by developing vaccinators to provide extension services in the communities. The following quote illustrates the enhanced uptake of nutrition extension services.

*‘Before we didn’t know […] we learned about why we should check-up while we are pregnant […] yes, I used to eat it [iron folic acid] also [received] from the government workers. We had a name [s]he [used to] come at home and give [iron tablet] every month.’* [BF2]

The data on their sustainability suggests mixed results. According to a few beneficiaries, health extension services provided at the grassroots level did not continue but were accessible for some at health centres. Regarding livestock extension, although a small number of implementers said that livestock vaccination services are continuing in some areas, some other implementers and beneficiaries reported to have stopped the community-based vaccination services or that beneficiaries receive it from sub-district headquarters. A beneficiary said:

*‘Before, [] was coming here who got training from Chalna. She gave vaccines to the duck and hens few days. Now she doesn’t give. Now it has been closed.’* [BM5]

### Women’s empowerment

The project improved financial access for women, as some beneficiaries made money selling vegetables, chickens, ducks and eggs. An implementer noted,

*‘They [women] were doing something that opened up more income. […] they were able to contribute to their families, which was empowerment in our understanding. We cannot say whether they can keep their income to themselves or not.’* [ID3]

While women’s access to selling and income was evident, they had less control over financial decision-making on where to spend the money. A few participants also mentioned that the decision-making depended upon the amount of money, as women could decide if the amount was small. Several women often used the earned money to address the need of their children (e.g. education) rather than their own. Decision-making, for some women, was the role of their mother-in-law or other household members, as indicated in the following quote.

*‘Taking a decision, in a family, is not possible for us, […] because we have father and mother-in-law, husbands. But I have a husband, so he takes the decision.’* [BF2]

The overall effect on gender equality was inconclusive. An implementer stated that the project contributed to gender equality by involving women in aquaculture, but gender-unequal practices were still evident. For example, women were generally responsible for housework, whereas men were mobile outside the households, for instance, to visit the market.

### Combination of the pathways and mechanisms leading to nutrition outcomes

Several beneficiaries benefitted from a combination of these pathways, which illustrates merit in the synergies among these to enhance nutrition outcomes, particularly through improved food consumption. The combinations reported for thirteen beneficiaries in the SSI were four (for five beneficiaries), followed by five (for three beneficiaries), two (for three beneficiaries) and three (for two beneficiaries). The synergy was also well reflected in the FGD. Leaders of the FFS group and trainers in the women’s farmers’ group reported a combination of multiple pathways compared with other beneficiaries

For some beneficiaries, a combination of *food production, nutrition-related knowledge and income* improved dietary and care practices. Increased poultry ownership led to egg consumption by women and children. Beneficiaries also began adding green leaves and eggs to food prepared for children or pregnant women. (Production) income and knowledge also played a significant role when there was no household production of specific food items. The combined role of knowledge and (production) income on dietary practices is visible through food-related expenditure. Several beneficiaries mostly spent on the food unavailable at the house, for instance, rice, potato, salt, chilli and oil. There were fewer responses to the expenditure on nutrient-rich foods such as vegetables, meat, milk and fish. The pathways that led to the spending on nutrient-rich food were knowledge, production and income, as indicated in the following quotes.

*‘The spinach and vegetables which are there, we bought them. […] Because there is nutrition. […] In some spinach, there is vitamin A. […] there is vitamin C.’* [ BF1]

*‘Consumed amaranth leaves with koi fish and cabbage fry [as lunch]. I bought cabbage from the market and spinach from home. [.] We caught fish from the stream.’* [BM2]

A couple of beneficiaries also reported expenses on highly processed and packaged foods for their children despite knowledge of the harms of consuming such foods because of the insistence of their children.

Nutrition-related knowledge and behaviour change and the strengthening of the local institutions also contributed to care practices such as breastfeeding, handwashing and strengthening of nutrition-specific actions (IFA supplementation during pregnancy). The pathway to appropriate breastfeeding practices emerged from enhanced knowledge. Handwashing practices appeared through improved knowledge and training on tipi-tap, a locally made handwashing station, as well as access to sanitation and handwashing facilities contributed by other projects. Pregnant women’s access to nutrient supplementation (IFA) was mainly due to the strengthening of institutions that enhanced the delivery of nutrition-specific actions.

The project’s contribution to empowering women through improving financial access, accompanied by increased income and nutritional knowledge, assisted some beneficiaries in nutrition-related purchases.

## Discussion

This mixed-methods research enabled us to assess the impact of the NSA interventions of the Integrated Agriculture and Health Based Interventions (IAHBI) project on improved nutrition and examined the pathways that led to the effects during the project funding cycle and the ones sustained 3 years after the funding period.

The IAHBI project significantly reduced underweight among children under 5 years but did not demonstrate a change in anaemia, stunting, or wasting. Significantly, there was a decrease in anaemia among lactating women, likely attributable to IFA supplementation during pregnancy. The reduction in anaemia among children was NS. A previous study from Nepal also showed no significant decline in anaemia among homestead food production groups and only a marginal effect when micronutrient power was added^(24)^. The significant decline in anaemia in lactating women observed at the endline, underscoring the importance of prenatal Fe supplementation as a key factor in reducing anaemia^(37)^. Past reviews also reported a limited impact of NSA on stunting or wasting^(2,3)^. The lack of effect on stunting could be due to a shorter project duration of 3 years, with only 2 years between the baseline and endline. As the risk of stunting starts during the first 1000 d, particularly in foetal life^(5)^, a longer duration beyond 3 years may demonstrate an effect on stunting^(2)^. The lack of reduction in wasting could be related to food shortages and illnesses over time^(38)^. This highlights that addressing stunting and wasting may require interventions that simultaneously address the longer-term and underlying causes of these conditions^(3)^.

The IAHBI project had varying effects on different aspects of infant and young child feeding. The significant increase in practices of early initiation of breastfeeding and exclusive breastfeeding is a notable effect, which might have been possible due to multiple interventions that integrated the production pathway with knowledge of IYCF and nutrition-specific interventions targeted to pregnant and lactating women. Despite an increase in household and women’s dietary diversity, the decrease in the proportion of children receiving minimum diet diversity was unexpected^(29)^. The decline in children’s MDD despite reduced underweight indicates that the project may have led to more stable food sources and thus more amount of food consumption, but less diversity. This could also be due to the high consumption of processed and ultra-processed food by children; the processed and ultra-processed food consumed by Bangladeshi children are mainly carbohydrates^(39)^. A past study using the same case also highlights that children preferred processed foods and drinks high in sugar and fats despite mothers’ knowledge of the disadvantages of consuming such foods^(19)^. As seen in South Africa, mothers’ reliance on limited food groups rather than diverse ones^(40)^ may have contributed to reduced dietary diversity in children. Further research is needed to understand why children’s MDD declined in this project and to guide targeted interventions. Strengthening social behaviour change communication and combining multiple pathways^(3)^ like food production and income generation could improve children’s diets, as our findings emphasise that changing nutrition outcomes often result from a combination of factors.

Access to water, sanitation and hygiene is a significant concern in Bangladesh. The project contributed to improved drinking water sources and sanitation facilities. However, the lack of access to water has further limited optimal WASH practices. Increased access to water, improved quality of water and sanitation facilities and enhanced implementation of hygiene interventions^(41)^ are critical to prevent the transmission of infection that may result in undernutrition.

As recommended by a past study^(3)^, we confirm that NSA should recognise the interplay between multiple pathways. In the case of the IAHB Project, the pathways that led to the effects on nutrition during the implementation were mainly food production, nutrition-related knowledge and strengthening of local institutions.

Although the income pathway was not focused like the production and nutrition-related knowledge/social behaviour change communication, NSA should invest in it, as resource limitations are a major barrier to adopting nutrition practices^(19)^. Past studies have also reported either one or more of these pathways on food production, nutrition-related knowledge and/or agricultural income^(1–3,42,43)^. Although existing evidence on income pathways focuses on selling^(42,43)^, this pathway should also consider cost-saving from reduced food purchases. Consistent with past research, beneficiaries, however, did not prioritise income for nutrition, although some expenses were recorded^(27)^. Given the significant barrier posed by resource constraints, future projects should address the full trajectory from increasing income to translating it into nutrition-related spending. In subsistence-based system, people do not rely solely on agricultural production. Therefore, future projects should diversify livelihoods to enhance income through alternative means, including agriculture (e.g. processing, employment, catering at mid-day meals^(19)^ and non-agriculture opportunities.

We also highlight the importance of strengthening the local institutions and women’s empowerment pathways. The strengthening of local institutions increased access to nutrition-specific services and agricultural extension for livestock. These contributed to the nutrient intake and food production pathway, respectively. Although the project did not directly envision women’s empowerment, some changes were reported. The positive change in financial decision-making by women – albeit on relatively small amounts of money through increased selling of food products – demonstrates some changes in women’s empowerment. However, the translation of raised income on nutrition-related expenditure was not clearly evident. A lack of nutrition-related expenditure could be because of limited control over the revenues, despite some control on selling^(44)^ or prioritisation of other needs due to poverty.

The *sustainability* of interventions’ effects on the pathways varied. Beneficiaries revealed production practices, especially of the vegetable and nutrition-related knowledge post-funding cycle. Consistent with the findings from an enhanced homestead food production program implemented in Burkina Faso^(45)^, we found the sustainability of livestock, especially poultries, is less, which could be due to either diseases or difficulties in adaptation to climatic conditions^(44)^ such as adaptation to saltwater. A good understanding of nutrition-related topics after the funding period illustrates the sustainability of the knowledge. Nevertheless, it could also be the case that the beneficiaries received knowledge from other initiatives implemented after the project. Although our data do not provide conclusive evidence on income post-funding period, later projects that integrated income and/or market, such as the National Agriculture Technology Program, may have further enhanced the pathway. Concerning women’s empowerment, it is worthwhile to provide gender-transformative approaches to change power relations. Sustaining the strengthened local institutions is challenging in Bangladesh because of inadequate capacity and limited human resources, especially in the Ministry of Livestock and Fisheries^(11)^. Future projects should prioritise the mechanisms to sustain the enhanced institutional capacity and service delivery developed during implementation^(19)^.

Pathways from NSA interventions to nutrition outcomes are complex. The complexity owes to both the nature of the interventions and the complex factors in the implementation area. Such factors include insufficient production due to inadequate land, a lack of resources, adverse geo-climatic and seasonal conditions, such as natural disasters and salinity. Addressing the barriers may require an improved income focused on women^(15)^, enhanced access to the market and improved production of diverse foods. A past study also recommended synergistic efforts that integrate farm diversity, diversify income, enhance the market and empower women, are required to improve dietary diversity in Bangladesh^(15)^. NSA projects also need to acknowledge facilitators such as other programmes, primary income sources beyond agriculture and existing community resources, to name a few.

The multisectoral collaboration between the agriculture and health sectors to deliver integrated interventions was an important strategy the project adopted. Various nutrition policies, such as the National Food and Nutrition Security Policy Plan of Action (NFNSP 2021–2030) and the second National Plan of Action for Nutrition (2016–2025), have recognised the importance of multisector collaboration. The project approach of integrating nutrition messages into agricultural demonstrations, supported by government extension workers^(19)^, aligns closely with Bangladesh’s 8th 5-year plan, emphasising basic nutrition integration into agricultural extension services^(46)^ as well as resonates with the objectives of the National Agriculture Policy. On the ground, however, the continued collaboration between sectors was mostly confined to the specific project’s scope^(19)^. The implementation of the policies at the grassroots level could benefit from increased coherence across projects by utilising platforms created by previous initiatives, such as the FFS^(19)^.

While these interventions show promise for scaling up, they necessitate fundamental changes in agricultural extension institutions at various levels to enhance their technical and financial capacities. This requires contextualisation based on different settings and continuous adaptation through participatory approaches. Actions may include enhancing accountability in the health and agriculture sectors^(47)^, empowering subnational institutions to decide on nutrition matters^(47)^ and integrating nutrition into agricultural staff job descriptions and training curricula^(18)^. However, as observed in Nigeria, solely having training curricula may not be sufficient, as effective delivery can be hindered by factors like heavy workloads, lack of motivation and limited resources^(48)^, some of which have also been identified in this project and requires addressing these challenges. These efforts should be accompanied by sufficient time to work with communities and enhance their capacity. Future funding initiatives should focus on identifying scalable interventions and developing mechanisms for their sustainability beyond the project period. As reported in another paper^(19)^, future interventions should prioritise mechanisms that support adoption beyond the funding cycle, considering both the capacity of implementing institutions and the beneficiaries involved. Given the substantial support from numerous NGO in Bangladesh, greater coherence and collaboration across multiple projects can contribute to implementation and improve intervention outcomes^(19)^.

There are two strengths of this study. First, it is the first study to holistically examine the impact of NSA interventions and the pathways during and after the project funding cycle. Second, the use of mixed methods enhanced the robustness of the result. However, four limitations may have influenced the findings. First, secondary quantitative data was used, and the evaluation lacked a comparison group, making it difficult to determine the causality between interventions and nutritional outcomes^(6)^. Inconsistent data collection for household and female food diversity in baseline and endline surveys hindered comparisons. Furthermore, as part of the surveys, MDD was measured using seven food groups, excluding breastmilk; this approach is clarified in the methods section for better interpretation. Second limitation is regarding information bias, which may have been caused due to a long recall period between implementation and post-implementation data collection and the transfer of participants to a different location. The third limitation is limited data on the complete aspects of the pathways. Women’s empowerment data focused on financial empowerment, and the information on gender equality was collected for only at one span of time 3 years after the end of the project cycle. Nonetheless, these limitations are cautiously discussed in the results. Future research should, therefore, gather data at two stages, during and after the project funding cycle.

### Conclusions

The IAHBI Project reduced underweight children and anaemic lactating women, improved DDS of households and women, increased MUAC scores and improved WASH practices. However, children’s MDD was slightly reduced, and wasting and stunting did not change significantly. Key pathways were food production, nutrition-related knowledge and strengthening local institutions, as these were the main focus of the project. The income and women’s empowerment pathways could be more effective in projects where these are intentionally integrated. While the sustainability of the knowledge and food production pathways was prominent, the continuation of strengthened local institutions was less evident. We stress the need to improve children’s dietary diversity, enhance the income–expenditure and women empowerment pathways and sustain livestock production as well as the capacity of local institutions to deliver nutrition-related health and agriculture interventions. Furthermore, these pathways are complex and non-linear, often reinforce and interact with each other and are influenced by various contextual factors. Future studies should examine sustained effects and pathways by collecting data during and after the project funding cycle. We also suggest considering the factors that enable or hinder the implementation and sustainability.

## Supporting information

Sharma et al. supplementary material 1 Sharma et al. supplementary material

Sharma et al. supplementary material 2 Sharma et al. supplementary material

Sharma et al. supplementary material 3 Sharma et al. supplementary material

## Footnotes

1 A simple handwashing station made of locally available materials such as plastic containers and jerry cans.

## Supplementary material

For supplementary material accompanying this paper, visit https://doi.org/10.1017/S1368980025000394
