# Supplementary material for: Pathways from integrated agriculture and health-based interventions to nutrition: a case from Southern Bangladesh
Source: Public Health Nutr. 2025 Aug 29;28(1):e133. doi: 10.1017/S1368980025000394 (PMC12465066; doi:10.1017/S1368980025000394)
Supplement: Sharma et al. supplementary material 1 — Sharma et al. supplementary material [file S1368980025000394sup001.docx]

## **Supplementary file 1. Variables included in the survey and the variables included in this study**

| **Variables included in the baseline and endline survey** | **Variables included in the secondary data analysis of this study** |
| --- | --- |
| - Household agricultural practices: horticulture, aquaculture, livestock and poultry - Household cooking techniques - Household food preservation - Water, hygiene and sanitation, and kitchen and food hygiene - Food consumption score and household dietary questionnaire - Salt consumption - Lactating women’s dietary diversity - Infant and young children feeding - Maternal care and nutrition - Nutritional status of children measured through anthropometry measurements | - Water, hygiene and sanitation: access to an improved sanitation facility, access to an improved drinking water source, purification of drinking water, and handwashing with soap - Household dietary diversity - Lactating women’s dietary diversity - Infant and young children feeding-early initiation of breastfeeding, exclusive breastfeeding and dietary diversity - Maternal care and nutrition: pregnant women’s dietary diversity, access to antenatal care, and intake of iron-folic acid during pregnancy - Nutritional status of children   - measured through anthropometric status: stunting, wasting and underweight   - micronutrient status-haemoglobin level and anaemia - Nutrition status of pregnant and lactating women measured through:   - underweight using mid-upper arm circumference (MUAC)   - micronutrient status, anaemia, using Hb, and Hb levels |
