# Supplementary material for: Pathways from integrated agriculture and health-based interventions to nutrition: a case from Southern Bangladesh
Source: Public Health Nutr. 2025 Aug 29;28(1):e133. doi: 10.1017/S1368980025000394 (PMC12465066; doi:10.1017/S1368980025000394)
Supplement: Sharma et al. supplementary material 2 — Sharma et al. supplementary material [file S1368980025000394sup002.docx]

## **Supplementary file 2. Summary of benefits as reported by the beneficiaries**

| **Topics/sectors** | **Training** |
| --- | --- |
| ***Agriculture*** | |
| Horticulture: vegetables and fruits | Training on cultivation using the bed, preparation and application of pesticides and fertilizers  Inputs: vegetable seeds (5-7 varieties), fertilizers, hand tools for weeding, fruits sapling (7 varieties) |
| Livestock | Training on livestock rearing (poultry, goat, sheep, cow), including shelter construction, food preparation and feeding, identification of diseases, seeking veterinary services (medicine), vaccination support  Inputs: poultry (5 pairs of ducks or hens), sheep or goat to women from least economic status, feeds, shelter (bamboo, *teen-*metal roof), vaccination by creating vaccinators |
| Aquaculture | Training on cultivation (discourage fishing when water is not clean, cleaning water using lime, cultivation of better-quality fish, proper light and shadow balance), food preparation and feeding, preparation and application of anti-pesticides using mustard seeds oil cake and rice powder, prevention of germs  Inputs: fish fingerlings/minnow (5 varieties), fish feed |
| ***Nutrition and WASH*** | |
| Topics (sessions/IEC materials) | Diverse food consumption (including sources of nutrients), nutrition requirement during childhood, pregnancy, lactation and adolescence, food preparation techniques, nutrient deficiency diseases, WASH |
| Cooking demonstration | Hands-on training on cooking diverse nutritious foods, food preparation and feeding to children and pregnant women (fish balls, jawl, omelettes, rice powder with sugarcane mixed and semolina, etc.), demonstration of proper cooking techniques  Inputs: cooking utensils to women’s groups and foods for demonstration (including rice, pulse, eggs) |
| Food preservation | Food preservation techniques (pickle, jelly and jam making), drying vegetables and fish, and seed drying to consume as legumes  Inputs: drying machines provided to groups |
| WASH facilities | Demonstration of handwashing station using locally available materials-*tipi tap* |
| Enhance uptake of nutrition-specific actions | Use of/referral to antenatal/postnatal services. Door to door distribution of iron-folic and/or counselling |
