# Supplementary material for: Pathways from integrated agriculture and health-based interventions to nutrition: a case from Southern Bangladesh
Source: Public Health Nutr. 2025 Aug 29;28(1):e133. doi: 10.1017/S1368980025000394 (PMC12465066; doi:10.1017/S1368980025000394)
Supplement: Sharma et al. supplementary material 3 — Sharma et al. supplementary material [file S1368980025000394sup003.docx]

**Supplementary file 3 Selected quotes on the pathways**

| **Pathways** | **Quotes** |
| --- | --- |
| Food production | *Before the project, we were also planting vegetables but in smaller quantities because we were not familiar with the bed system and lacked knowledge of proper fertilizer application [BF1].* |
|  | *After receiving training and seeds, we began planting by creating small beds in limited spaces, allowing us to grow food for home consumption. This home cultivation has become a significant source of income because we no longer need to purchase food with money [BF1].* |
|  | *I no longer have ducks as they died due to illness. However, it's important to clarify that we do have 1 or 2 ducks that are offspring from the ducks provided by the project [BF1].* |
|  | *Within our group, the 8 individuals who received inputs sold all the hens and ducks they acquired but did not pursue hatchery activities further. My hen sometimes weighs 8 kg (not received from the project) because I follow the project's guidance by feeding rice curry, taking them to the community clinic, and providing special care that others do not [B_L2]* |
| Agriculture  Income | *The project came up with so many nice things after which, beneficiaries are farming vegetables in a planned way, using organic fertilizers, avoiding the random use of pesticides, and all these things ultimately sum up to more profit [ID6]* |
|  | *After receiving training and seeds, we began planting by creating small beds in limited spaces, allowing us to grow food for home consumption. This home cultivation has become a significant source of income because we no longer need to purchase food with money [BF1].* |
|  | *As long as the project was there and the hens and ducks lay eggs that time they ate. They ate and also, they sold them. Like they were giving 12 ducks. [B L2]* |
| Knowledge on nutrition and WASH | *My child was around 2 years old when the project began. I learned that by age 2, their brain development is crucial. We were advised to provide them with extra meals six times a day [BF1].* |
|  | *We were taught the importance of proper handwashing with soap before eating and after using the toilet to prevent the spread of germs and diseases like diarrhoea, dysentery, and cholera. We diligently follow these practices [BF1].* |
|  | *I received vegetables and hens from the project. Due to the high cost of eggs (10 taka each) and the nutritional needs of my children (requiring one egg daily along with spinach and vegetables), we raised ducks and hens at home to meet their dietary needs. Eggs are a valuable source of energy and contribute to their studies, among other reasons for raising them [BM8].* |
| Women’s empowerment | *Aquaculture activities are predominantly carried out by men, largely influenced by religious beliefs that restrict women from engaging in outdoor activities like aquaculture. While women are typically involved in horticulture and poultry, we aim to empower women by teaching them aquaculture skills (ID9).* |
|  | *In village settings, women often stay at home while men handle market activities such as selling and purchasing. However, women are becoming increasingly self-reliant and are making decisions independently, a significant change facilitated by the project. Previously, women had limited knowledge of earning and spending money, but now they contribute to family finances alongside men (ID2).* |
| Strengthening service delivery | *During the project, a health extension worker used to visit homes and give iron pills monthly [BF2].* |
|  | *[Name deleted for anonymity], who received training from Chalna, used to come here and give vaccines to the ducks and hens. However, this practice has now stopped [BM5].* |
